# Intragenomic Conflict between Knob Heterochromatin and B Chromosomes Is the Key to Understand Genome Size Variation along Altitudinal Clines in Maize

**DOI:** 10.3390/plants10091859

**Published:** 2021-09-08

**Authors:** Graciela Esther González, Lidia Poggio

**Affiliations:** Departamento de Ecología, Genética y Evolución, Instituto de Ecología, Genética y Evolución (IEGEBA, Consejo Nacional de Investigaciones Científicas y Técnicas—CONICET)—Laboratorio de Citogenética y Evolución (LACyE), Facultad de Ciencias Exactas y Naturales, Universidad de Buenos Aires, Ciudad Autónoma de Buenos Aires 1428, Argentina; lidialidgia@yahoo.com.ar

**Keywords:** altitudinal clines, B chromosomes, genome size, heterochromatin, intragenomic conflict, maize, nucleotype

## Abstract

In maize, we studied the causes of genome size variation and their correlates with cultivation altitude that suggests the existence of adaptive clines. To discuss the biological role of the genome size variation, we focused on Bolivian maize landraces growing along a broad altitudinal range. These were analyzed together with previously studied populations from altitudinal clines of Northwestern Argentina (NWA). Bolivian populations exhibited numerical polymorphism for B chromosomes (Bs) (from 1 to 5), with frequencies varying from 16.6 to 81.8 and being positively correlated with cultivation altitude. The 2C values of individuals 0B (A-DNA) ranged between 4.73 and 7.71 pg, with 58.33% of variation. The heterochromatic knobs, detected by DAPI staining, were more numerous and larger in individuals 0B than in those with higher doses of Bs. Bolivian and NWA landraces exhibited the same pattern of A-DNA downsizing and fewer and smaller knobs with increasing cultivation altitude, suggesting a mechanistic link among heterochromatin, genome size and phenology. The negative association between the two types of supernumerary DNA (knob heterochromatin and Bs), mainly responsible for the genome size variation, may be considered as an example of intragenomic conflict. It could be postulated that the optimal nucleotype is the result of such conflict, where genome adjustment may lead to an appropriate length of the vegetative cycle for maize landraces growing across altitudinal clines.

## 1. Introduction

The knowledge of genome dynamics is essential for understanding the diversification of DNA content among closely related taxa. The genus *Zea* shows a high variation in nuclear DNA content, with the C-value differing between species, subspecies, populations, inbred and open pollination lines under contrasting growing conditions. On this basis, *Zea mays* ssp. *mays* (maize) represents an interesting model to address general questions concerning the sources of genome size variation and its ecological and evolutionary consequences. 

Maize grows in a broad range of agro-ecological areas across the American continent. A large number of landraces (>300) are cultivated from the lowlands to the highlands in the Andean region, which is one of the most important centers of maize diversity [1,2]. In particular, more than 60 morphological native landraces have been described in northern Argentina [3], with a variability comparable to that observed for Bolivian landraces growing at a wide altitudinal range [4,5]. A high variability in DNA content has been reported for many maize inbred lines and landraces from the American continent [6,7,8,9,10,11]. The underlying causes of genome size variation are important to understand its biological role. In maize, it has been attributed to differences in the heterochromatin located in the knobs and interspersed DNA (e.g., retrotransposon families), which make up over 70% of the nuclear genome [10,11,12,13]. Knobs are conspicuous heterochromatic regions located in sub-terminal positions on the chromosomes of maize [2]. DAPI staining allows revealing the chromosome location of the knobs. Their variation in number, size and *tandem* repeats composition has been used to characterize maize landraces [10,11].

Studies on American maize populations have reported that knob number and heterochromatin percentage are positively related to genome size. In addition, the DNA content was found to be negatively correlated with the latitude and altitude of cultivation [8,9,10,14,15] and positively correlated with the generation time [10,16]. A recent study involving temperate and tropical maize inbred lines grown at sea level revealed that DNA content increases progressively with delayed flowering time [17]. All these results suggest that genome downsizing may be associated with the rapid vegetative growth and early flowering observed in short growing seasons, which are typical of cool highland regions [6,8,11,18,19]. This assumption is supported by evidence showing that the percentage of knob heterochromatin of the A-chromosome set (A-HC) is negatively correlated with cultivation altitude, but positively correlated with the vegetative period. Realini et al. [10,20] found a positive correlation between the length of the vegetative cycle and A-HC in maize landraces from Northeastern Argentina (NEA). This relationship was also reported for maize populations from different localities [15,21,22]. The length of the vegetative cycle was proposed to be optimized through artificial selection for an appropriate percentage of heterochromatin [10,15,20].

The numerical polymorphism of B chromosomes (Bs) is also an important factor contributing to genome size variation in maize [2,8,9,11,18,23]. Bs are non-essential, harmful and parasitic chromosomes which lack homology with any member of the normal complement (the A-chromosome set), but possess mechanisms of drive which enhance their transmission rates by several processes of non-mendelian inheritance [24,25,26]. Moreover, the presence of Bs was associated with some phenotypic effects, suggesting that they are not genetically inert. These effects are usually cumulative, depending on the number but not on the presence or absence of Bs [27]. Recent studies in maize showed that Bs contain active genes and can express miRNAs by altering the transcription of A genes and affecting the expression of A-derived miRNAs [28,29]. As selfish entities, Bs of maize act like a “genomic sponge” which collects and maintains sequences of diverse origin that may insert, spread and amplify in these chromosomes [27]. 

Polymorphism for presence and doses of Bs was reported in maize native populations from Northwestern Argentina (NWA). Several studies found a significant negative correlation between the mean number of Bs and the percentage of knob heterochromatin of the A-chromosome set (A-HC) [8,9,11]. These authors postulated that in populations with lower A-HC content, Bs might be maintained at higher frequencies so as to preserve an optimal nucleotype. The term nucleotype *sensu* Bennett defines the conditions of the nucleus that affect cellular and developmental parameters such as chromosome size, nuclear volume, cellular volume, mitotic cycle time, duration of meiosis and minimum generation time [30,31].

In maize, the increasing evidence that genome size, percentage of heterochromatin and frequency of Bs are correlated with altitude/latitude of cultivation strongly supports the existence of ‘adaptive clines’ [15].

The present study investigates the causes of genome size variation and its possible relationship to organismal characteristics and environmental parameters. For this purpose, we focused on Bolivian maize landraces cultivated along an altitudinal cline from 200 to 3250 m.a.s.l. These were analyzed together with previously reported landraces from altitudinal clines of NWA to discuss the biological role of genome size variation. We propose the existence of an intranuclear conflict between the knob heterochromatin and the presence of B chromosomes. 

## 2. Results

**Figure 1:**
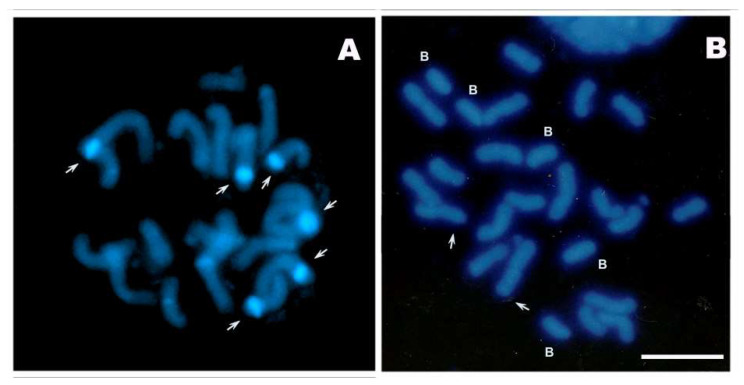
DAPI staining of mitotic metaphases. (**A**) Individual 0B (Duro Amazónico landrace). (**B**) Individual with 5 Bs (Pisankalla landrace). Ref.: white arrows show heterochromatic knobs (DAPI-positive bands); B letter indicates the B chromosomes. Bar: 10 µm.

We performed chromosome counts on 547 individuals belonging to 12 populations of seven Bolivian landraces that grow from 200 to 3250 m.a.s.l. (Table 1). Populations showed variability in the number (from 1 to 5) and frequency (from 16.6 to 81.8) of Bs, with higher means and frequencies of Bs for populations cultivated at higher than at lower altitudes (Table 1). In Figure 1, the DAPI stained mitotic metaphases illustrates the B chromosomes and the heterochromatic knobs (DAPI-positive bands). The knobs were more numerous and larger in individuals 0B than in those with higher doses of Bs.

Mean DNA content of 120 plants of Bolivian maize landraces cultivated at different altitudes are shown in Table 2. The 2C values of individuals 0B (A-DNA) ranged between 4.73 and 7.71 pg, with 58.33% of variation (Table S1). Mean A-DNA values ranged between 5.26 and 6.23 pg, with 18.44% of variation (Table 2). The landraces Duro Amazónico (200 m.a.s.l.) and Tiumuru (3200 m.a.s.l.) exhibited the highest and the lowest 2C mean A-DNA values, respectively. We also estimated the 2C values of individuals having from 1 to 3 doses of Bs (Table 2 and Table S1).

**Figure 2:**
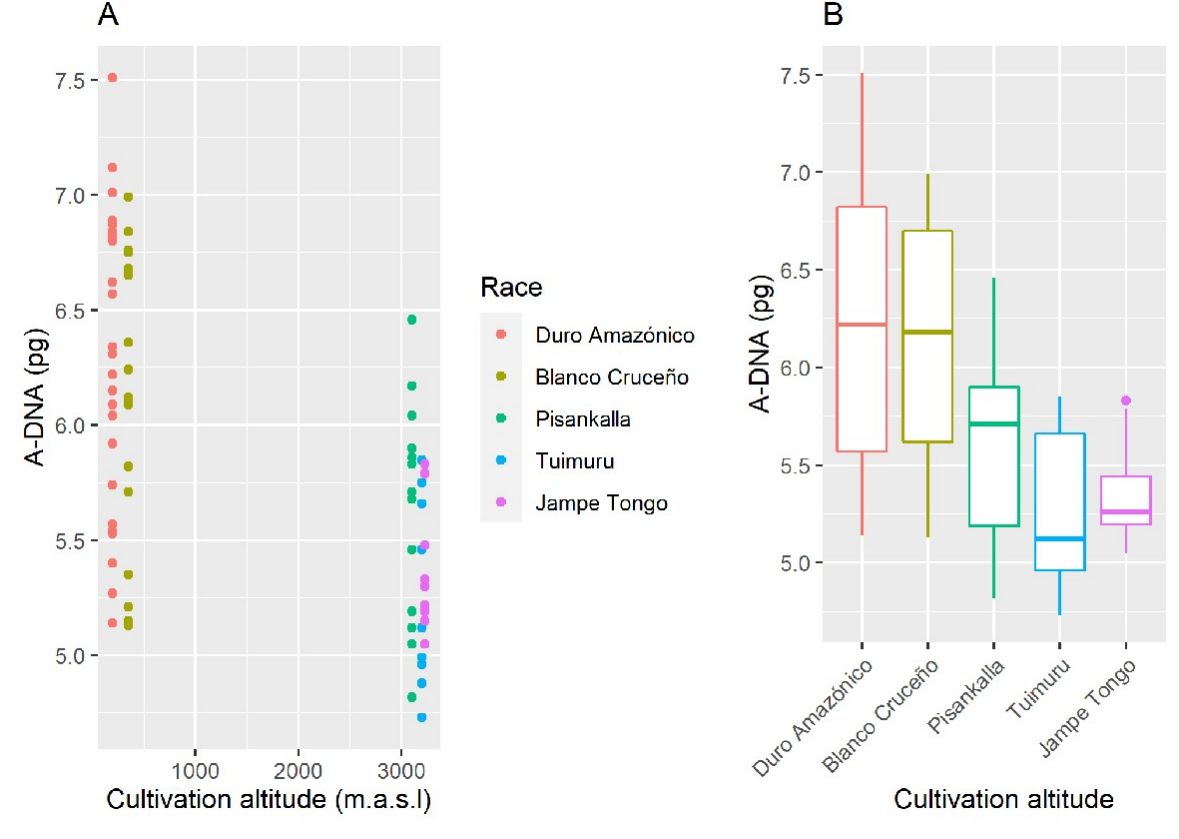
A-DNA content vs. cultivation altitude in Bolivian maize landraces. (**A**) Dispersion graph. (**B**) Boxplots. Data from Table S1.

DNA content of individuals 0Bs (A-DNA) data were grouped by altitudes of cultivation, <700 m.a.s.l. (Duro Amazónico and Blanco Cruceño landraces) and >3000 m.a.s.l. (Pisankalla, Tiumuru and Jampe Tongo landraces), since intermediate altitudes were not sampled. *T*-test showed significant difference between both groups (t = −6.04, *p* < 0.001, α = 0.05), i.e., populations at higher altitudes have lower A-DNA content than those growing at lower altitudes (Figure 2). 

**Figure 3:**
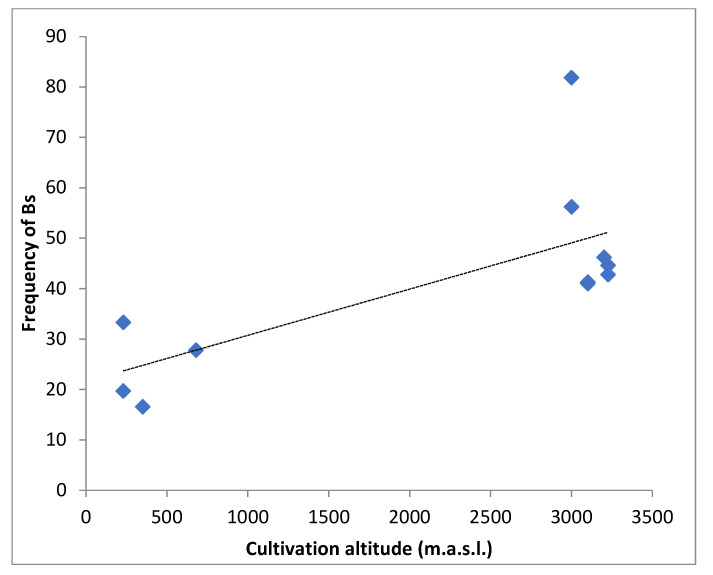
Frequency of B chromosomes vs. cultivation altitude in Bolivian maize landraces.

**Figure 4:**
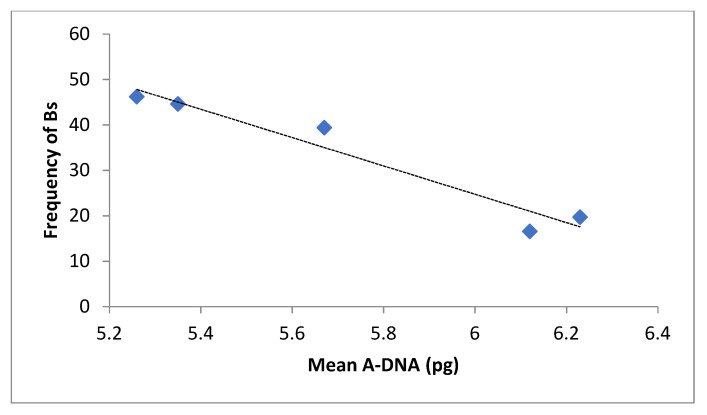
Frequency of B chromosomes vs. mean A-DNA in Bolivian maize landraces.

The frequency of Bs data were also grouped by altitudes (<700 and >3000 m.a.s.l.) and the *T*-test showed significant differences between both groups (t = 4.62, *p* < 0.0007, α = 0.05). The frequency of Bs was higher in populations growing at more than 3000 m.a.s.l. (Figure 3). Figure 4 shows that the frequency of Bs decreases as the mean A-DNA content increases (r = −0.67, *p* = 0.218).

**Figure 5:**
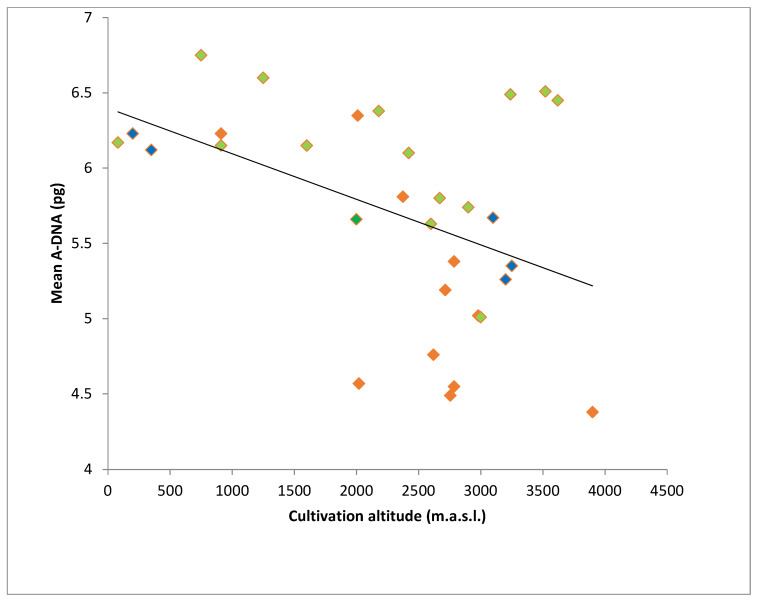
Mean A-DNA vs. cultivation altitude in Bolivian and NWA maize landraces. Blue: Bolivian landraces. Green: NWA landraces from Rosato et al., 1998. Red: NWA landraces from Fourastié et al., 2017 (data from Table S2).

The mean A-DNA 2C value of the landraces from Bolivia and of two different altitudinal clines from NWA [9,11] were significantly and negatively correlated with the altitude of cultivation (r = −0.46, *p* = 0.01) (Figure 5, Table S2).

## 3. Discussion

A wide variability in nuclear DNA content has been observed for different maize inbred lines and landraces from the American continent. In Northwestern Argentina (NWA), one of the southernmost areas of native maize cultivation, the DNA content of the A-chromosome complement (A-DNA) showed a difference of 40% between the minimum and the maximum 2C values [8,11,15]. Our results revealed an even greater variation of 58.33% for the Bolivian landraces growing along a broad altitudinal cline (from 200 to 3250 m.a.s.l.), with the A-DNA 2C value ranging between 4.73 and 7.71 pg.

The variation in total DNA content is mainly caused by the intraspecific variation in the abundance of transposable elements and number and size of heterochromatic knobs [11,12,13,22,32]. Relationships observed in previous studies between intraspecific variations in DNA content and phenotypic, cytological and/or environmental characteristics suggest the existence of ‘adaptive clines’ and that the genome size plays a biological role [8,11,15,16,32].

In agreement with several works reporting a negative relationship between total DNA content and altitude/latitude of cultivation of maize, landraces from NWA growing along a wide altitudinal cline (from 80 to 3900 m.a.s.l.), showed a clear downward trend of genome size with increasing cultivation altitude [8,9,11]. The same trend was observed in the Bolivian landraces here analyzed. Based on these results, it was postulated that genome downsizing would be associated with rapid vegetative growth and early flowering time. These characteristics are typical of populations adapted to shorter growing seasons in highland regions [8,15,19,20,21,22,32]. Variation in total DNA content, comprising both genic and non-genic DNA (nucleotype [30]) affects cellular and developmental parameters. Therefore, selection has been proposed to act either directly or indirectly on ‘multiple phenotypes’ under different environmental conditions along an altitudinal cline [11,15,31].

The analysis of the cytological parameters involved in genome size variation is necessary to understand the relationship between DNA content and environmental and phenological characteristics. Maize presents intrapopulation (polymorphism) and interpopulation (polytypism) variation in heterochromatin amount, which is mainly located in knobs. The variation in number, chromosomal position and size of knobs were used for the cytogenetic characterization of local varieties of maize from Argentina [10,14,33,34]. In maize populations, the heterochromatin amount has shown to be positively correlated with A-DNA content and negatively correlated with cultivation altitude [8,9,11,15]. In addition, the mean knob number proved to be negatively correlated with cultivation latitude or altitude in several maize populations across America [8,9,11,15]. The Bolivian and the NWA landraces exhibited the same pattern of decreasing A-DNA content and number of knobs with increasing cultivation altitude.

The positive relationships between heterochromatin percentage and A-DNA content and their negative correlations with cultivation altitude so far reported for all the studied landraces, suggest a mechanistic link among heterochromatin, genome size and phenology [9,10,11,20]. Realini et al. [10,20], who studied maize populations from NEA, found that heterochromatin percentage is positively correlated with the length of the vegetative period. The authors interpreted the variation in heterochromatin percentage and DNA content as the result of artificial selection on flowering time. Jian et al. [22] observed a moderate positive correlation between genome size and 180-bp knob sequence abundance in tropical and temperate inbred maize lines. They also found that genome size was associated with the flowering time and proposed that selection for breeding materials with earlier flowering times can be assisted by choosing germplasms with smaller genome sizes. All these data support the hypothesis that selection against the number and size of knobs leads to altitudinal genome downsizing [11,15].

Another important source of variation in total DNA content is the presence/absence of Bs. The Bolivian landraces studied here exhibited numerical polymorphism for Bs, which ranged from 1 to 5, with their frequencies varying from 16.6 to 81.8. Likewise, the NWA landraces presented 1 to 8 Bs per plant, with 1, 2 and 3 being the predominant doses [8,9,11]. The NWA populations showed the numerical polymorphism for Bs along the altitudinal cline and a higher frequency over ca. 2000 m.a.s.l. In addition, the frequency of Bs was negatively correlated with the number of heterochromatic knobs and heterochromatin percentage [11]. Negative associations between the occurrence of Bs and the number of knobs have also been reported for North American, Indian and Italian populations (revisited in [8]). In the Bolivian landraces, individuals without Bs had a larger number and size of knobs, which were identified as DAPI-positive bands on mitotic metaphases. On the other hand, chromosomes from individuals with a higher dose of Bs had fewer and smaller knobs. Higher-altitude landraces (3100 m.a.s.l.), such as Pisankalla and Valle Alto, had a smaller number of knobs and shorter life cycles (from 74 to 98 days) than did lower-altitude landraces (200-350 m.a.s.l.), such as Amazónico and Blando Cruceño (life cycle: from 112 to 132 days) [4,5].

To analyze the relationship among Bs, DNA content and altitude of cultivation it is interesting to point out that the Bs of the samples from Bolivia and NWA showed a similar size and morphology, suggesting that they made a comparable contribution to total DNA content [9,11,14, present work]. In agreement with other studies, a significant difference was found between the frequency of Bs, being higher in populations that grow above 3000 m.a.s.l. than in those that grow below 700 m.a.s.l. Theoretically, the addition of either heterochromatic knobs or Bs should increase the genome size of an individual and Ayonoadu and Rees [35] calculated that each B resulted in an increase in total DNA content of about 5%. However, the situation becomes more complex for natural populations because of polymorphisms in both heterochromatic knobs and the presence of Bs. Indeed, some authors proposed that the fraction of DNA corresponding to Bs may be masked due to polymorphism of heterochromatic knobs in A-chromosomes [8,9,11]. They also suggested that the negative association between Bs and heterochromatic knobs would allow the maintenance of an optimum nucleotype. Moreover, the variable downsizing of A-DNA content along with the doses of Bs would be influenced by the genotypical make-up of the landraces [11].

Although Bs are regarded as selfish entities, molecular markers used for the NWA landraces suggest that the altitudinal cline of Bs is maintained by selective forces [36]. An in-depth knowledge of the structure, function and transmission of the Bs may shed light on their possible adaptive role. Huang et al. [28] found that maize Bs contain active genes that alter the transcription of A genome genes and that their impact is enhanced by increasing Bs doses. B chromosomes are enriched with genes involved in their own maintenance as well as in several important biological functions and are likely to influence the nuclear environment affecting the function of other chromatin regions [29,37,38]. Based on all these considerations, it is valid to postulate that the inverse relationship between Bs and heterochromatin found in NWA and Bolivian landraces, indicates the presence of an intranuclear conflict between the knob heterochromatin of the A chromosomes (A-HC) and the Bs. The association between Bs and supernumerary heterochromatic segments has been examined in different organisms such as grasshoppers, crickets and plants [39,40,41,42,43,44]. In maize, Rhoades and Dempsey [45] found that knobbed regions of the A complement are often eliminated at the second division of microspores having two or more Bs and proposed a mechanism of heterochromatin loss induced by B chromosomes. The existence of intragenomic conflict was observed between the TR-1 and 180-bp knob repeats of maize, which operate in competition with each other in the presence of other drive genes [46]. 

To explain the inverse relationship between knob heterochromatin and number of Bs in maize landraces from NWA, it has been proposed that there may be a maximum limit to the mass of nuclear DNA (nucleotype), so that Bs would be tolerated as long as this maximum limit is not exceeded [9,11]. The data discussed in the present study allow us to reinforce that the negative association between the two types of supernumerary DNA (knob heterochromatin and Bs) may be considered as an example of intragenomic conflict in maize. The notion that genetic and/or epigenetic changes occur in repeated DNA sequences due to interaction between knob heterochromatin and Bs improves our understanding of the concept of optimal nucleotype. In this regard, it could be postulated that the optimal nucleotype would be the result of the intragenomic conflict between the A-heterochromatin and B chromosomes, where rapid genome adjustment between repetitive and selfish elements may lead to an appropriate length of the vegetative cycle for maize landraces across altitudinal clines.

## 4. Materials and Methods

### 4.1. Plant Material

Maize seeds were collected and provided by the Germplasm Bank of Centro de Investigaciones Fitoecogenéticas de Pairumani, Cochabamba, Bolivia. The cultivation altitude and accession numbers of the 13 studied populations, belonging to 7 landraces, are listed in Table 1. 

### 4.2. Cytological Studies

Seeds were germinated at 28 °C for 2–3 days in wet paper. The primary root tips were excised, pre-treated with 0.002 M 8-hydroxyquinoline at room temperature for 3 h, fixed in 3:1 absolute ethyl alcohol: acetic acid solution for 24 h and stored in 70% ethanol at −20 °C until use. Fixed root tips were washed in 0.01 M citric acid–sodium citrate buffer (pH 4.6) to remove fixative and digested in 2% cellulase (Onozuka R10, Merck, NJ, USA) and 20% pectinase (Sigma-Aldrich, St. Louis, MO, USA) at 37 °C for 45 min. Then, they were squashed on a slide with a drop of acetic acid 45 % (v v^−1^). Cover slides were removed by freezing and the preparations were air-dried until use.

Cytological preparations were stained with a drop of DAPI (4′-6-diamino-2-phenylindole, 2 µg/mL) and incubated in darkness at room temperature for 20 min. The slides were washed gently with 4 X saline-sodium citrate buffer (SSC, 3 M NaCl, 0.3 M sodium citrate, pH 7), mounted with a drop of Vectashield (Vector Lab., Burlingame, CA, USA) anti-fade solution and covered with a glass coverslip. Slides were examined by fluorescence microscopy (Axiophot; Carl Zeiss, Oberkochen, Germany). 

Chromosome counts were made on more than 500 individuals belonging to 13 populations from 7 Bolivian landraces. The number (or dose) of B chromosomes (Bs) in each individual was determined from mitotic metaphase preparations. For each population, the mean number of Bs was estimated by averaging the sum of B doses among the individuals over the total number of individuals examined. Likewise, the frequency of Bs was calculated as the number of individuals having Bs over the total number of individuals studied.

### 4.3. Feulgen Staining and Cytophotometry

The fixed root tips were stained as described by Tito et al. [7]. The amount of Feulgen staining per nucleus, expressed in arbitrary units, was measured at a wavelength of 570 nm with the scanning method with a Zeiss Universal Microspectrophotometer (UMSP 30; Carl Zeiss, Oberkochen, Germany). The DNA content (2C-value) expressed in picograms (pg) was calculated using *Allium cepa* var. Ailsa Craig as a standard (2C = 33.55 pg, [47]). DNA content of 120 plants was estimated. At least 20 telophase nuclei (2C) per plant and 22–30 plants per population were measured.

The estimation of DNA content in individuals without B chromosomes (0B) was referred to as A-DNA content. The variation between the highest and lowest A-DNA content values was estimated as: [(2Cmax − 2Cmin) * 100/2Cmin].

### 4.4. Statistical Analysis

The software R version 4.0.2 was applied, considering α = 0.05.

## Figures and Tables

**Table 1:** Number of individuals with different doses, mean and frequency of B chromosomes in Bolivian maize populations.

Landrace	Population Voucher	Cultivation Altitude (m.a.s.l.)	0B	1B	2B	3B	4B	5B	Total indiv.	Mean of Bs	Frequency of Bs
Duro Amazónico	BOZM0724	200	23	7	6	2	0	0	38	0.65	39.40
Duro Amazónico	BOZM0723	230	73	16	2	0	0	0	91	0.22	19.70
Blando Amazónico	BOZM1050	230	20	8	1	0	1	0	30	0.46	33.30
Blando Cruceño	BOZM0715	350	70	14	0	0	0	0	84	0.16	16.60
Blando Cruceño	BOZM1421	680	13	4	0	1	0	0	18	0.39	27.80
Pisankalla	BOZM0038	3000	4	9	7	2	0	0	22	1.32	81.80
Tuimuru	BOZM0678	3000	7	7	2	0	0	0	16	0.69	56.20
Pisankalla	BOZM0962	3100	23	10	4	1	0	1	39	0.66	41.02
Pisankalla	BOZM0684	3100	37	20	5	1	0	0	63	0.52	41.30
Tuimuru	BOZM0672	3200	29	15	6	4	0	0	54	0.72	46.29
Jampe Tongo	BOZM0192	3225	31	14	10	1	0	0	56	0.66	44.60
Jampe Tongo	BOZM0786	3250	12	3	5	1	0	0	21	0.76	42.85
	Total		353	129	50	13	1	1	547	0.49	

**Table 2:** Mean DNA 2C-values (pg) of individuals with different doses of B chromosomes in Bolivian maize landraces.

Landrace	Cult. Altitude (m.a.s.l.)	0B (±S/D)	1B (±S/D)	2B (±S/D)	3B (±S/D)
Jampe Tongo-BOZM0192	3250	5.35 ± 0.25	5.88 ± 0.07	6.30 ± 0.07	6.45 ± 0.11
Tuimuru-BOZM0672	3200	5.26 ± 0.39	5.08 ± 0.22	5.68 ± 0.69	6.43 ± 0.05
Pisankalla-BOZM0684	3100	5.67 ± 0.49	5.58 ± 0.41	6.14 ± 0.30	6.63 ± 0.93
Blando Cruceño-BOZM0715	350	6.12 ± 0.63	6.59 ± 0.79	---	---
Duro Amazónico-BOZM0724	200	6.23 ± 0.64	6.57 ± 0.38	---	---

## Data Availability

Data are contained within the article or supplementary material.

## Supplementary Materials

The following are available online at https://www.mdpi.com/article/10.3390/plants10091859/s1. Table S1: Individual DNA 2C-values (pg) in Bolivian maize landraces. Table S2: Mean A-DNA in Bolivian and NWA maize populations.
