# Supplementary material for: Intragenomic Conflict between Knob Heterochromatin and B Chromosomes Is the Key to Understand Genome Size Variation along Altitudinal Clines in Maize
Source: Plants (Basel). 2021 Sep 8;10(9):1859. doi: 10.3390/plants10091859 (PMC8468181; doi:10.3390/plants10091859)
Supplement: Supplementary file 1 [file plants-10-01859-s001.zip › plants-1311577-supplementary.pdf]

Supplementary Material

Table S1. Individual DNA 2C-values (pg) in Bolivian maize landraces.

**Landrace**	**Cultivation altitude** m.a.s.l.	**DNA (2C)** ± SD	**2n**	**Media DNA** ± SD
Tuimuru BOZM0672	3200	5.85 ± 0.64	20	
		5.75 ± 0.34	20	
		4.88 ± 0.84	20	
		4.99 ± 0.78	20	
		4.73 ± 0.43	20	
		4.96 ± 0.74	20	
		5.12 ± 0.66	20	
		5.46 ± 1.14	20	
		5.66 ± 0.36	20	5.26 ± 0.39
		5.46 ± 0.35	20+1B	
		5.01 ± 0.42	20+1B	
		4.87 ± 0.58	20+1B	
		4.99 ± 0.69	20+1B	
		5.10 ± 0.43	20+1B	
		5.38 ± 0.37	20+1B	
		4.81 ± 0.29	20+1B	5.08 ± 0.22
		4.91 ± 0.40	20+2B	
		6.52 ± 0.69	20+2B	
		6.21 ± 1.42	20+2B	
		5.08 ± 1.14	20+2B	5.68 ± 0.69
		6.38 ± 0.31	20+3B	
		6.48 ± 0.35	20+3B	6.43 ± 0.05
Pisankalla BOZM0684	3100	6.46 ± 0.48	20	
		5.12 ± 0.36	20	
		5.90 ± 0.42	20	
		5.71 ± 0.35	20	
		5.46 ± 0.37	20	
		5.86 ± 0.63	20	
		6.04 ± 1.06	20	
		4.82 ± 1.10	20	5.67 ± 0.49
		5.83 ± 0.59	20+1B	
		5.68 ± 0.32	20+1B	
		5.19 ± 0.18	20+1B	
		5.05 ± 0.56	20+1B	
		6.17 ± 0.28	20+1B	5.58 ± 0.41
		6.26 ± 0.39	20+2B	
		6.12 ± 0.35	20+1B	
		5.91 ± 0.24	20+2B	
		6.60 ± 0.42	20+2B	
		5.62 ± 0.33	20+2B	
		6.33 ± 0.47	20+2B	6.14 ± 0.30
		5.29 ± 0.28	20+3B	
		6.33 ± 0.52	20+3B	
		7.82 ± 0.55	20+3B	
		7.08 ± 0.57	20+3B	6.63 ± 0.93
Jampe Tongo BOZM0192	3225	5.19 ± 0.26	20	
		5.05 ± 0.20	20	
		5.79 ± 0.55	20	
		5.21 ± 0.37	20	
		5.15 ± 0.42	20	
		5.83 ± 0.38	20	
		5.22 ± 0.58	20	
		5.30 ± 0.64	20	
		5.33 ± 0.36	20	
		5.48 ± 0.58	20	5.35 ± 0.25

		5.86 ± 0.42	20+1B	
		5.90 ± 0.38	20+1B	
		5.74 ± 0.22	20+1B	
		6.03 ± 0.62	20+1B	5.88 ± 0.10
		6.21 ± 0.15	20+2B	
		6.28 ± 0.64	20+2B	
		6.41 ± 0.36	20+2B	
		6.32 ± 0.53	20+2B	6.30 ± 0.07
		6.58 ± 0.14	20+3B	
		6.27 ± 0.78	20+3B	
		6.43 ± 0.35	20+3B	
		6.53 ± 0.84	20+3B	6.45 ± 0.11
Blanco Cruceño BOZM0715	350	6.84 ± 0.41	20	
		5.21 ± 0.61	20	
		6.99 ± 0.26	20	
		5.13 ± 0.56	20	
		5.71 ± 0.51	20	
		6.75 ± 0.74	20	
		6.36 ± 0.89	20	
		5.15 ± 0.30	20	
		5.82 ± 0.44	20	
		5.35 ± 0.27	20	
		5.14 ± 0.21	20	
		6.11 ± 0.80	20	
		6.66 ± 0.54	20	
		6.09 ± 0.29	20	
		6.65 ± 0.26	20	
		6.76 ± 0.54	20	
		6.75 ± 0.33	20	
		6.24 ± 0.37	20	
		6.12 ± 0.28	20	
		6.68 ± 0.45	20	6.12 ± 0.63
		7.24 ± 0.25	20+1B	
		5.79 ± 0.32	20+1B	
		5.82 ± 0.33	20+1B	
		7.53 ± 0.32	20+1B	6.59 ± 0.79
Duro Amazónico BOZM0724	200	6.34 ± 0.42	20	
		6.57 ± 1.12	20	
		5.14 ± 0.25	20	
		5.74 ± 0.23	20	
		6.04 ± 1.06	20	
		5.40 ± 0.34	20	
		6.31 ± 0.42	20	
		5.53 ± 0.98	20	
		5.54 ± 0.80	20	
		6.89 ± 0.74	20	
		7.51 ± 0.64	20	
		6.87 ± 1.29	20	
		6.22 ± 0.86	20	
		5.92 ± 0.31	20	
		5.57 ± 0.29	20	
		6.62 ± 0.35	20	
		5.54 ± 0.39	20	
		6,09 ± 0.68	20	
		6.15 ± 0.71	20	
		5.27 ± 0.87	20	
		6.80 ± 0.56	20	
		6.84 ± 0.37	20	
		6.82 ± 0.38	20	
		7.01 ± 0.51	20	
		7.12 ± 0.34	20	6.23 ± 0.64
		6.79 ± 0.29	20+1B	

	6.09 ± 0.34	20+1B	
	6.11 ± 0.66	20+1B	
	6.97 ± 0.35	20+1B	
	6.89 ± 0.43	20+1B	6.57 ± 0.38

Table S2. Mean A-DNA in Bolivian and NWA maize populations.

**Landrace**	**Cult. altitude** m.a.s.l.	**Mean A-DNA** pg
Garrapata 3*	3900	4.38
Altiplano**	3620	6.45
Altiplano 1**	3520	6.51
Jampe Tongo	3250	5.35
Harinoso**	3240	6.49
Tiumuru	3200	5.26
Pisankalla	3100	5.67
Altiplano**	3000	5.01
Pisingallo 3*	2980	5.02
Capia Rosado**	2900	5.74
Amarillo Grande 3*	2785	4.55
Garrapata 2*	2785	5.38
Amarillo Grande 2*	2755	4.49
Pisingallo 2*	2714	5.19
Blanco**	2670	5.80
Garrapata 1*	2620	4.76
Capia Blanco**	2600	5.63
Amarillo Grande**	2420	6.10
Pisingallo 1*	2373	5.81
Amarillo Grande 1*	2020	4.57
Blanco**	2180	6.38
Amarillo Chico**	2010	6.35
Amarillo Chico**	2000	5.66
Pisingallo**	1600	6.15
Blanco y 8 Rayas**	1250	6.60
Orgullo Cuarenton*	910	6.23
Orgullo Cuarenton**	910	6.15
Blanco y 8 Rayas**	750	6.75
Blanco Cruceño	350	6.12
Duro Amazónico	200	6.23
Pichingá**	80	6.17

*from Fourastié *et al*. 2017; **from Rosato *et al*. 1998.
